# Value of fat-suppressed T2-weighted imaging for predicting short-term pain relief after sclerotherapy for venous malformations in the extremities

**DOI:** 10.1007/s11604-023-01442-x

**Published:** 2023-05-12

**Authors:** Shuji Nagata, Norimitsu Tanaka, Asako Kuhara, Tomoko Kugiyama, Shuichi Tanoue, Masamichi Koganemaru, Yusuke Uchiyama, Kiminori Fujimoto, Toshi Abe

**Affiliations:** Department of Radiology, Kurume University School of Medicine, 67 Asahi-Machi, Kurume, Fukuoka 830-0011 Japan

**Keywords:** Magnetic resonance imaging, Fat-suppressed T2-weighted imaging, Venous malformations, Polidocanol sclerotherapy, Short-term pain relief

## Abstract

**Purpose:**

This study aimed to evaluate the value of fat-suppressed T2-weighted imaging (FS-T2WI) for predicting short-term pain relief after polidocanol sclerotherapy for painful venous malformations (VMs) in the extremities.

**Materials and methods:**

This retrospective study included patients with painful VMs in the extremities between October 2014 and September 2021, had their first sclerotherapy without history of surgical therapy, and underwent magnetic resonance imaging before sclerotherapy. Pain relief was assessed 2 months after 3% polidocanol sclerotherapy and was categorized as follows: progression, no change, partial relief, or free of pain. The associations between pain relief and imaging features on FS-T2WI were analyzed.

**Results:**

The study included 51 patients. The no change, partial relief, and free of pain groups included 6 (11.8%), 25 (49.0%), and 20 (39.2%) patients, respectively. No patient experienced progressive pain. The lesion diameter was ≤ 50 mm in 13 (65.0%) patients in the free of pain group, whereas it was > 50 mm in all patients in the no change group (p = 0.019). The lesions showed well-defined margin in 15 (75.0%) patients in the free of pain group, whereas they showed ill-defined margin in 5 (83.3%) patients in the no change group (p = 0.034). The most common morphological type was cavitary in the free of pain group (14 [70.0%] patients), whereas there was no patient with cavitary type lesion in the no change group (p = 0.003). Drainage vein was demonstrated in 6 (100%), 22 (88.0%), and 11 (55.0%) patients in the no change, partial relief, and free of pain group, respectively (p = 0.011).

**Conclusion:**

A lesion size of 50 mm or less, a well-defined margin, a cavitary type, and no drainage vein on FS-T2WI were significant features for predicting short-term pain relief after polidocanol sclerotherapy for painful VMs in the extremities.

## Introduction

Venous malformations (VMs) are part of the spectrum of vascular malformations and are the most common vascular malformations. VMs are characterized by slow blood flow and an abnormal venous network owing to errors in endothelial cell morphogenesis. They tend to grow, often painfully, in proportion to the growth of the patient and often enlarge during puberty and pregnancy [1]. VMs in the extremities often require pain relief, unlike most cases that involve the head and neck, which require treatment for cosmetic or functional recovery purposes.

Magnetic resonance imaging (MRI) is excellent for defining the extension of VMs and assessing their relationship with adjacent structures. In particular, fat-suppressed T2-weighted imaging (FS-T2WI) shows VMs and the veins continuous with VMs as structures with high signal intensity owing to stagnant or slow blood flow in abnormally dilated venous spaces [1–4].

Generally, symptomatic VMs are treated using multiple modalities, including surgery, sclerotherapy, and laser therapy, as well as compression therapy and medications, such as low-dose aspirin [5]. The emergence of sclerotherapy as a cost-effective and minimally invasive technique has spurred its use as either a monotherapy or in conjunction with surgery [6, 7]. Direct puncture venography (DPV) performed before sclerotherapy is the gold standard diagnostic tool when the findings in other imaging examinations are equivocal [5]. VMs have the following three main venographic patterns: cavitary, spongy, and dysmorphic [1]. Moreover, Puig et al. classified VMs based on anatomical and hemodynamic features into the following four types: type I, isolated VMs without peripheral drainage; type II, VMs that drain into normal veins; type III, VMs that drain into dysplastic veins; and type IV, VMs with venous ectasia [8]. They reported that sclerotherapy can be safely performed in patients with type I and type II, while patients with type III and type IV must be carefully considered [8].

There are several reports on the relationship between clinical outcomes after sclerotherapy and imaging features based on MRI [1, 9–15]. However, to our knowledge, there is no report on the prediction of pain relief after sclerotherapy for VMs in the extremities, using imaging features including presence of a drainage vein on FS-T2WI. Thus, this study aimed to evaluate the value of FS-T2WI for predicting short-term pain relief after polidocanol sclerotherapy for painful VMs in the extremities.

## Materials and methods

### Study approval and consent

Approval for this retrospective study was obtained from our institutional review boards. The requirement for informed consent was waived by the ethics committee owing to the retrospective nature of the study.

### Study population

Patients were included if they presented with painful VMs in the extremities between October 2014 and September 2021 at our hospital, had their first sclerotherapy session without history of surgical therapy, and underwent baseline MRI including FS-T2WI before sclerotherapy.

**Fig. 1:**
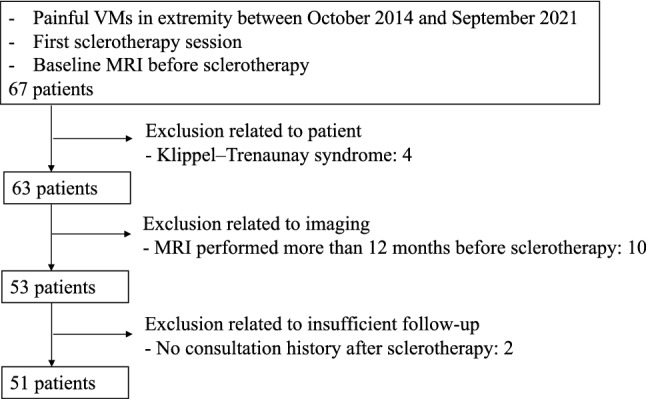
Study flowchart

A total of 67 consecutive patients were identified. Among them, 16 patients were excluded for the following reasons: VMs were part of Klippel–Trenaunay syndrome (4 patients), MRI was performed more than 12 months before sclerotherapy (10 patients), and consultation history was absent after sclerotherapy (2 patients) (Fig. 1).

#### Sclerotherapy

Sclerotherapy was performed under either local or general anesthesia. General anesthesia was chosen for pediatric patients. Direct puncture of the lesion was performed using a 23- or 25-gauge needle with or without ultrasound guidance. All VMs were diagnosed by confirming blood reflux and the findings of DPV, including blood pooling and a drainage vein. We added iodixanol (270 mg/mL iodine, Visipaque; GE Health Care, Chicago, IL, USA) to 3% polidocanol (Aethoxysklerol, Kreussler Pharma, Wiesbaden, Germany) to be visible during injection and to avoid leaking into the extra-venous surrounding tissue due to unstable punctured needle. The sclerosing foam was produced based on Tessari’s method, agitating 3% polidocanol, air, and iodixanol in a ratio of 2:4:1 by repeated transfer between two syringes [16]. We used relatively low ratio of air compared with the original Tessari's method which was used in the treatment of varicose veins because the higher ratio of polidocanol was considered necessary for the treatment of VMs, especially when the lesion was extensive.

The 3% polidocanol foam was injected under digital subtraction angiography (DSA) guidance until a sufficient amount of the form accumulated in the VMs. If a drainage vein appeared before sclerosing foam was pooled in the VMs, a tourniquet was applied at the proximal portion of the VMs on the extremity, or the drainage vein was pressed with fingers. The amount of polidocanol foam was determined according to the estimated volume of the VMs on DPV.

### Clinical analysis

Data were collected retrospectively using electronic patient records and a picture archiving and communication system (PACS). Clinical success was decided by two attending interventional radiologists by means of consensus, using electronic patient records. Pain was assessed at 2 months after sclerotherapy because swelling and pain can increase in the VM region for some time after the procedure. Pain outcomes were categorized as follows: progression, no change, partial relief, or free of pain. Pain Progression was defined as worsening of pain compared to that before sclerotherapy. No change in pain was defined as neither worsening nor improvement of pain compared to that before treatment. Free of pain was defined as the complete disappearance of pain. Patients who had neither no change in pain nor complete disappearance of pain were considered to have partial relief of pain.

### MRI examination

All examinations were performed using a 3.0-T MRI system (Magnetom Skyra; Siemens Healthineers, Erlangen, Germany) with adjustments of the coils, field of view, and matrix, depending on the lesion size, location, and depth. All MRI scans were performed within 12 months before sclerotherapy. MRI protocols had to include at least one T1-weighted fast spin-echo imaging and two planes (axial and coronal or sagittal) of T2-weighted fast spin-echo imaging with fat-suppression techniques (CHESS or Dixon). The ranges of repetition time and echo time were 500–700 ms and 10–15 ms for T1-weighted fast spin-echo imaging, and were 4000–5200 ms and 40–90 ms for T2-weighted fast spin-echo imaging, respectively. Section thickness ranged from 3 to 5 mm.

### Image analysis

Two board-certified radiologists independently evaluated the imaging findings of VMs on FS-T2WI. The radiologists were blinded to both the clinical information and outcomes after sclerotherapy. After each radiologist interpreted the images, they discussed any differences in their evaluations to reach a consensus. All VMs were evaluated for the longest lesion diameter, margin definition, morphological type, presence of a drainage vein, presence of a thrombus or phlebolith, and presence of a fluid–fluid level on FS-T2WI. The margin definition was categorized into well-defined and ill-defined. The morphological types of VMs were classified into three patterns—cavitary, spongy, and dysmorphic pattern—based on FS-T2WI. VMs with or without a few septa were considered to have a cavitary pattern, those with small honeycomb cavities were considered to have a spongy pattern, and those with dysmorphic veins were considered to have a dysmorphic pattern.

### Statistical analysis

Continuous data are presented as means with standard deviation (SD) or as medians with ranges or interquartile ranges (IQRs). Categorical data are presented as counts and percentages. The Kruskal–Wallis test with the Steel–Dwass test was used to compare pain outcome groups in terms of age and the longest lesion diameter. The association between pain outcome and age, sex, location, depth, lesion diameter category (≤ 50 mm or > 50 mm), and features on FS-T2WI (margin definition, morphological type, presence of a drainage vein, presence of a thrombus or phlebolith, and presence of a fluid–fluid level) were analyzed using the Fisher exact test.

The interobserver variation in the MRI interpretation data was analyzed using the kappa statistic [17] for the diagnosis made before agreement by consensus. The agreement was classified as follows: poor (κ = 0–0.20), fair (κ = 0.21–0.40), moderate (κ = 0.41–0.60), good (κ = 0.61–0.80), and excellent (κ = 0.81–1.00). All statistical tests were two-sided, and a *p* value < 0.05 was considered significant. All statistical analyses were performed using JMP version 16.0.0 (SAS Institute; Cary, NC, USA).

## Results

### Patients

The study included 51 patients (34 women; mean age ± SD, 25.8 ± 13.9 years) with VMs. The amount of 3% polidocanol ranged from 35 to 180 mg (mean ± SD, 85 ± 49.9 mg). Among the 51 patients, 40 (78.0%) experienced pain at the injection site after 3% polidocanol sclerotherapy. This early complication was self-limited, and it resolved with conservative approaches. No patient developed skin injury, soft tissue necrosis, local infection, or sensory loss.

In the clinical assessment, no patient had progressive pain after sclerotherapy. The no change, partial relief, and free of pain groups included 6 (11.8%), 25 (49.0%), and 20 (39.2%) patients, respectively. Pain outcomes were not significantly different by age (*p* = 0.36), sex (*p* = 0.77), location (*p* = 0.29), or depth (*p* = 0.25). On the other hand, the longest lesion diameter significantly differed among the three groups (*p* = 0.038). The longest lesion diameter was significantly larger in the no change group than in the free of pain group (*p* = 0.006). The longest lesion diameter was ≤ 50 mm in 13 (65.0%) patients in the free of pain group, whereas it was > 50 mm in all patients in the no change group (p = 0.019) (Table 1).

**Table 1:** Patient characteristics and pain outcomes

Characteristics	All patients (n = 51)	Pain outcome			p
		No change (n = 6)	Partial relief (n = 25)	Free (n = 20)	
Age (y), median (IQR)	23 (15–35)	31 (14–44)	22 (13–28)	27 (21–36)	0.36^a^
Sex					0.77^b^
Women	34 (66.7)	4 (66.7)	18 (72.0)	12 (60.0)	
Men	17 (33.3)	2 (33.3)	7 (28.0)	8 (40.0)	
Location					0.29^b^
Upper extremity	16 (31.4)	1 (16.7)	6 (24.0)	9 (45.0)	
Lower extremity	35 (68.6)	5 (83.3)	19 (76.0)	11 (55.0)	
Depth					0.25^b^
Subcutaneous	15 (29.4)	0 (0)	8 (32.0)	7 (35.0)	
Muscle	28 (54.9)	4 (66.7)	15 (60.0)	9 (45.0)	
Combined	8 (15.7)	2 (33.3)	2 (8.0)	4 (20.0)	
Longest lesion diameter (mm), median (IQR)	48 (25.2–77.2)	83.5 (71.7–130.7)	52 (26–115.5)	35 (21–58)	0.038^a^
≦50 mm	25 (49.0)	0 (0)	12 (48.0)	13 (65.0)	0.019^b^
> 50 mm	26 (51.0)	6 (100)	13 (52.0)	7 (35.0)	

Except where indicated otherwise, data are numbers of patients with percentage of patients in parentheses. *IQR* interquartile range

^a^Kruskal-Wallis test with the Steel–Dwass test was used to compare age and longest lesion diameter between pain outcome groups

^b^Fisher exact test was used to compare sex, location, depth, and size between pain outcome groups

### Interobserver agreement

Interobserver agreement was good (κ = 0.62–0.77) for margin definition, morphological type, presence of a drainage vein, and presence of a fluid–fluid level, and was moderate (κ = 0.55) for presence of a thrombus or phlebolith (Table 2).

**Table 2:** Appearance of VMs on FS-T2WI at each pain outcome

	All patients (n = 51)	κ^a^	Pain outcome			p^b^
			No change (n = 6)	Partial relief (n = 25)	Free (n = 20)	
Margin definition		0.62 (0.41–0.82)				0.034
Well-defined	30 (58.8)		1 (16.7)	14 (56.0)	15 (75.0)	
Ill-defined	21 (41.2)		5 (83.3)	11 (44.0)	5 (25.0)	
Morphological type		0.77 (0.61–0.93)				0.003
Cavitary	27 (52.9)		0 (0)	13 (52.0)	14 (70.0)	
Spongy	16 (31.4)		4 (66.7)	10 (40.0)	2 (10.0)	
Dysmorphic	8 (15.7)		2 (33.3)	2 (8.0)	4 (20.0)	
Drainage vein		0.75 (0.54–0.95)				0.011
Absence	12 (23.5)		0 (0)	3 (12.0)	9 (45.0)	
Presence	39 (76.5)		6 (100)	22 (88.0)	11 (55.0)	
Thrombus or phlebolith		0.55 (0.28–0.81)				0.79
Absence	26 (51.0)		4 (66.7)	13 (52.0)	9 (45.0)	
Presence	25 (49.0)		2 (33.3)	12 (48.0)	11 (55.0)	
Fluid–fluid level		0.71 (0.47–0.94)				0.44
Absence	38 (74.5)		4 (66.7)	21 (84.0)	13 (65.0)	
Presence	13 (25.5)		2 (33.3)	4 (16.0)	7 (35.0)	

Except where indicated otherwise, data are numbers of patients with percentage of patients in parentheses

^a^Interobserver agreement was assessed using the kappa statistic. Data presented in parentheses are 95% CIs

^b^Fisher exact test was used to compare the appearance of VMs between pain outcome groups

### Image analysis

Table 2 indicates that three of the five individual features (i.e., margin definition, morphological type, and drainage vein) on FS-T2WI were significantly different among the groups.

**Fig. 2:**
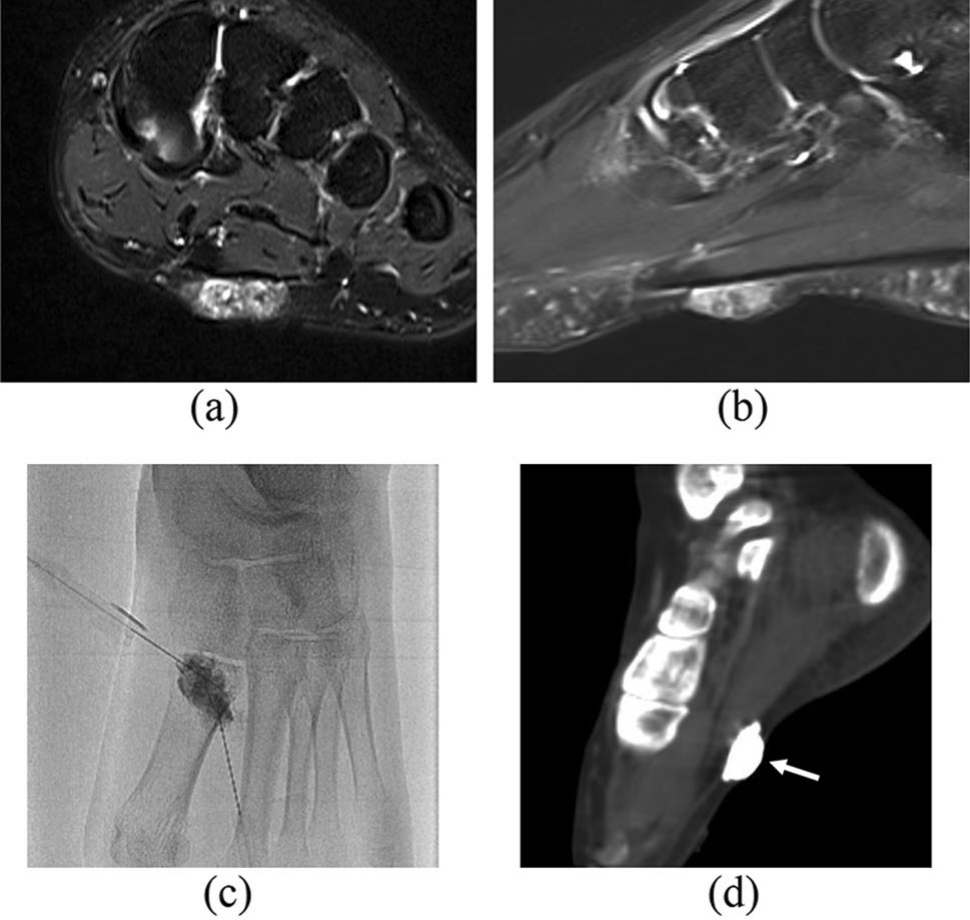
Case of a 28-year-old woman with a VM in her left foot. Axial **a** and sagittal, **b** FS-T2WI images show a hyperintense well-defined and cavitary lesion. The lesion measures 18 mm in the longest diameter. A drainage vein is absent on FS-T2WI.** c** A direct puncture venogram shows no visualization of a drainage vein. **d** After 3% polidocanol sclerotherapy, a cone beam CT image demonstrates dense pooling of contrast medium without a drainage vein (arrow). The clinical outcome was “free of pain”

**Fig. 3:**
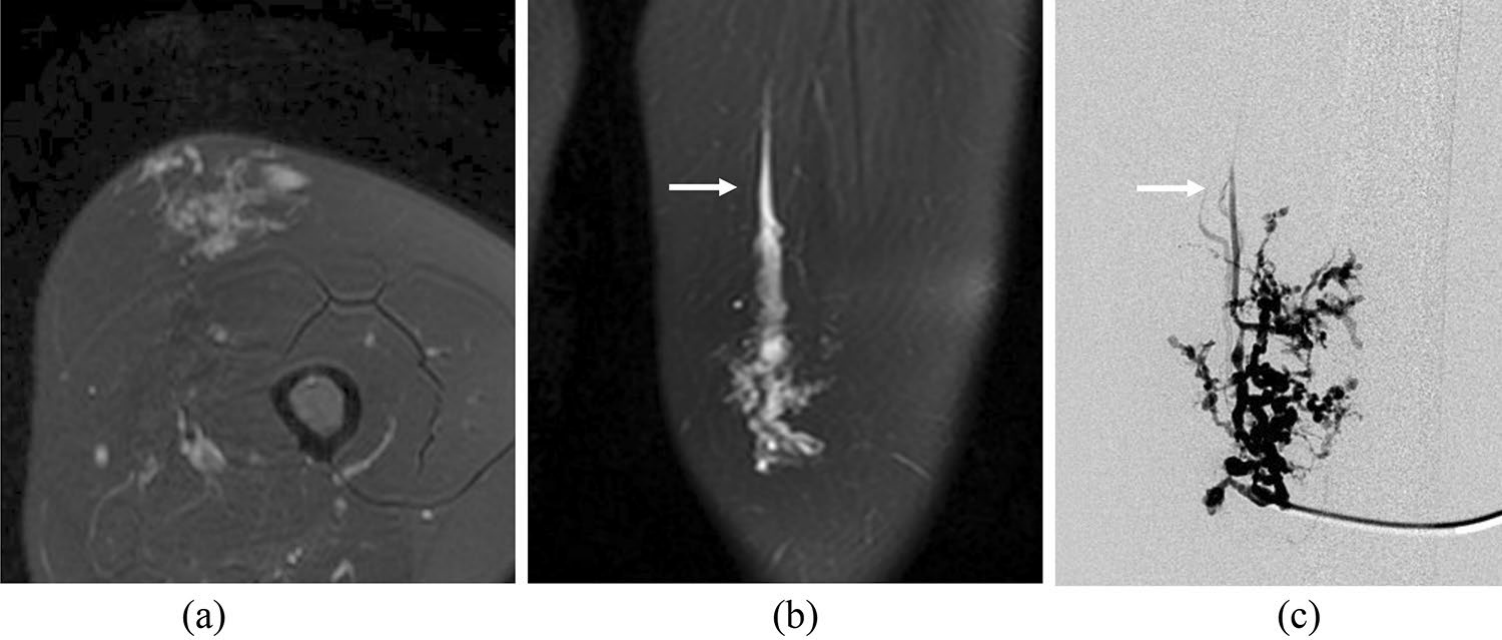
Case of a 17-year-old woman with a VM in her left thigh. Axial (**a**) and coronal (**b**) FS-T2WI images show a hyperintense ill-defined and dysmorphic lesion with a drainage vein (arrow). The lesion measures 43 mm in the longest diameter. **c** A digital subtraction angiography image demonstrates contrast medium draining from the VM into a drainage vein (arrow). The clinical outcome was “partial relief of pain”

VMs had well-defined margin in 30 out of 51 patients (58.8%). The pain outcome was significantly different by the margin definition (*p* = 0.034). The lesion showed well-defined margin in 15 (75.0%) patients in the free of pain group, whereas it showed ill-defined margin in 5 (83.3%) patients in the no change group. Cases of VMs with well- and ill-defined margins are shown in Figs. 2 and 3, respectively.

The most common morphological type was the cavitary type (52.9%), followed by the spongy type (31.4%) and dysmorphic type (15.7%). The pain outcome was significantly different by the morphological type (*p* = 0.003). The most common type was cavitary in the free of pain group (14 [70.0%] patients), whereas there was no patient with cavitary type lesion in the no changes group.

**Fig. 4:**
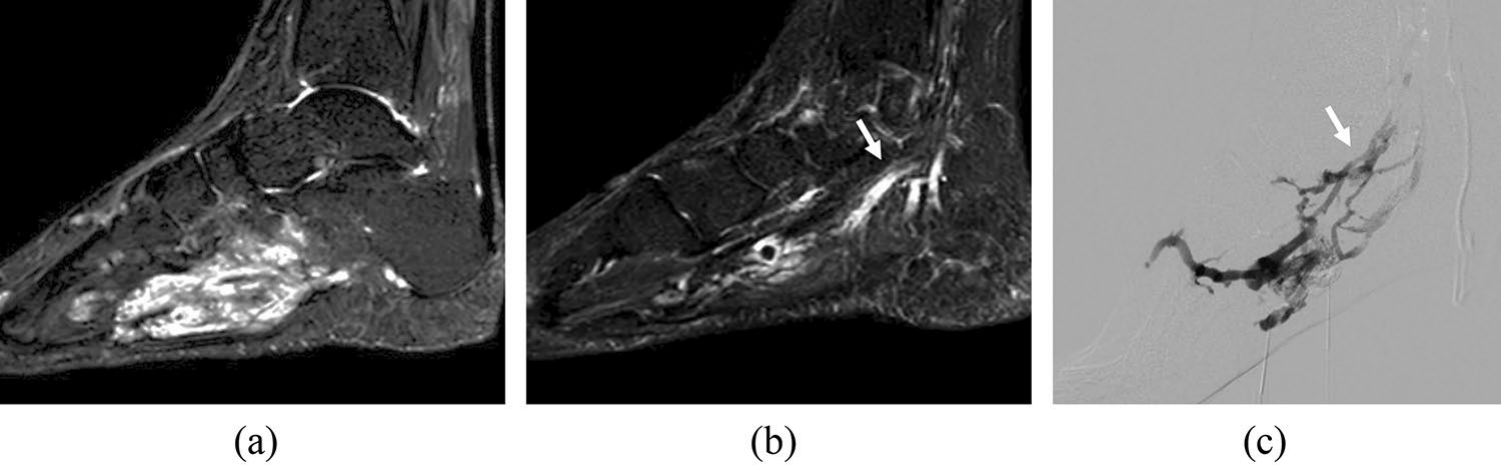
Case of a 38-year-old woman with a VM in her left foot. **a**,** b** Sagittal FS-T2WI images show a hyperintense, ill-defined, and spongy lesion with a dilated drainage vein (arrow). The lesion measures 89 mm in the longest diameter. **c** A digital subtraction angiography image demonstrates contrast medium draining from the VM into a drainage vein (arrow). The clinical outcome was “no change in pain”

A drainage vein was present in 39 out of 51 patients (76.5%) (Figs. 3, 4). The pain outcome was significantly different according to the presence of a drainage vein (*p* = 0.011). Drainage vein was demonstrated in 6 (100%), 22 (88.0%), and 11 (55.0%) patients in the no change, partial relief, and free of pain group, respectively.

There was no significant difference in the pain outcome according to the presence of a thrombus or phlebolith (*p* = 0.79), or the presence of a fluid–fluid level (*p* = 0.44).

## Discussion

This study evaluated the value of FS-T2WI to predict the effect of polidocanol sclerotherapy on painful VMs in the extremities. We found that a small size, a well-defined margin, a cavitary type, and no drainage vein on FS-T2WI were important factors for predicting pain relief after treatment. Our results may help physicians become familiar with the potential successes and limitations of polidocanol sclerotherapy according to imaging findings on FS-T2WI and may help in determining appropriate candidates for the treatment.

Regarding size, sclerotherapy was more effective for VMs less than 50 mm (longest diameter) than those greater than 50 mm. VMs with well-defined margins on FS-T2WI tended to more frequently be associated with the complete disappearance of pain after sclerotherapy. These results are consistent with the findings of previous reports [10, 14, 15, 18]. We believe that smaller lesions are more likely to respond to sclerotherapy than larger lesions owing to larger contact area with polidocanol.

With regard to the morphological type, the cavitary type tended to show a preferable response, whereas the spongy type tended to show a limited to poor response. We speculate that the spongy type VMs were divided by the septa into the small parts which had sparse communication with each other, and the sclerosant was less likely to spread throughout the lesion. Dubois et al. classified VMs into cavitary, spongy, and dysmorphic types based on DPV and found that the results with sclerotherapy were better for the cavitary and dysmorphic types than the spongy type [1]. Although the imaging modality was different (i.e., FS-T2WI rather than DPV), our results supported the findings of this previous report.

Regarding the drainage vein, pain relief tended to be associated with the absence of a drainage vein on FS-T2WI, whereas no pain relief tended to be associated with the presence of a drainage vein. Yun et al. evaluated the relationship between the drainage vein and treatment efficacy using DPV and reported that no or delayed visualization of the drainage vein was associated with a preferable response to percutaneous sclerotherapy [10]. Mimura et al. reported that the group with good stasis of polidocanol during sclerotherapy showed better pain relief than the group with poor stasis of the sclerosant, and the efficacy of sclerotherapy is dependent upon the time and surface area of contact with the endothelium [15]. Drainage veins demonstrated on FS-T2WI would suggest that sclerosants may not be retained in VMs during sclerotherapy, negatively affecting the pain relief after treatment.

There have been reports on the prediction of the therapeutic effects of sclerotherapy for VMs using dynamic contrast-enhanced MRI [12, 19]. Xia et al. evaluated the value of dynamic contrast-enhanced MRI focusing on hemodynamic characterization for predicting the effectiveness of pain reduction, cosmetic improvement, and functional improvement of sclerotherapy [12]. They concluded that the maximum intensity time ratio (MITR) was a predictor of the response to sclerotherapy, and a high MITR value was indicative of unsatisfactory outcomes. Unlike their study, we did not use contrast media for MRI. The advantages of non-contrast MRI include cost-effectiveness, free of side effects, and short examination time.

This study has three major limitations. First, because this was a retrospective study, semi-quantitative assessment using the Visual Analog Scale (VAS) was not available. The evaluation of pain after sclerotherapy had to rely solely on information available in the electronic medical records. Second, we assessed degree of pain relief at 2 months after sclerotherapy. That is, only short-term pain improvement of VMs was evaluated, but their effectiveness in the long-term was not demonstrated in this study. Third, we focused on pain relief and did not consider functional limitations and cosmetic satisfaction in this study. Nakahara et al. evaluated the efficacy of percutaneous sclerotherapy for VMs in pediatric patients, using overall satisfaction, including not only pain but also swelling, functional limitations, and cosmetic disfigurement, assessed with a self-assessment questionnaire. They concluded that sclerotherapy is effective for relieving symptoms and many of the patients were satisfied with the outcomes [20]. Further investigations, including those of functional limitations and cosmetic satisfaction, are needed to determine the value of FS-T2WI before sclerotherapy.

In conclusion, a lesion size of 50 mm or less, a well-defined margin, a cavitary type, and no drainage vein on FS-T2WI before sclerotherapy were significant features in predicting short-term pain relief after polidocanol sclerotherapy for painful VMs in the extremities.
